# Kaposiform Hemangioendothelioma with Kasabach–Merritt Phenomenon in a Neonate: A Case Report

**DOI:** 10.1055/a-2699-8104

**Published:** 2025-09-19

**Authors:** Yuyang Zheng, Rongjie Wu, Qilin Chen, Shiyu Xiong, Jialu Yun, Wei Peng

**Affiliations:** 1 The First Clinical Medical College of Gannan Medical University, Ganzhou, Jiangxi, People's Republic of China; 2 Department of Pediatric Surgery, First Affiliated Hospital of Gannan Medical University, Ganzhou, Jiangxi, People's Republic of China

**Keywords:** kaposiform hemangioendothelioma, Kasabach–Merritt phenomenon, sirolimus, neonate, thrombocytopenia

## Abstract

**Background:**

Kaposiform hemangioendothelioma (KHE) is a rare, aggressive vascular tumor frequently complicated by Kasabach–Merritt phenomenon (KMP), a life-threatening consumptive coagulopathy. Neonatal KHE-KMP management requires urgent intervention but is complicated by immunosuppression risks, especially in Bacillus Calmette–Guérin (BCG)-vaccinated infants.

**Methods:**

A full-term male neonate with prenatal right upper limb thickening presented postnatally with a violaceous, firm mass. Laboratory findings confirmed KMP. Due to recent BCG vaccination, sirolimus was initially withheld. First-line therapies failed, prompting sirolimus initiation on day 3, supplemented by fibrinogen transfusions.

**Results:**

Platelets normalized by day 13 (283 × 10 ^9^ /L) with marked tumor regression. Transient fever/diarrhea resolved with supportive care. At discharge (day 27), platelets stabilized (183 × 10 ^9^ /L). Three-month follow-up showed sustained platelet recovery (268–532 × 10 ^9^ /L), near-complete tumor resolution, and age-appropriate development. Prophylactic trimethoprim-sulfamethoxazole prevented infections.

**Conclusion:**

Sirolimus is a critical salvage therapy for refractory neonatal KHE-KMP, even in BCG-vaccinated infants. Timely initiation reverses life-threatening coagulopathy and achieves favorable outcomes, necessitating multidisciplinary monitoring to balance immunosuppression risks.

**Importance for the Pediatric Surgeon**

This case reinforces sirolimus as a lifesaving salvage therapy for refractory neonatal KHE-KMP. Pediatric surgeons must advocate for early sirolimus initiation, coordinate multidisciplinary care, and prioritize coagulopathy stabilization over theoretical infection risks in critical settings.

## Introduction

Kaposiform hemangioendothelioma, a rare borderline vascular tumor with an estimated incidence of 0.91 per 100,000, 1 predominantly manifests during infancy. A subset of cases may develop severe thrombocytopenia and consumptive coagulopathy, triggering the Kasabach–Merritt phenomenon, a life-threatening complication associated with high mortality if untreated. This study retrospectively analyzes the diagnostic and therapeutic management of a neonate with kaposiform hemangioendothelioma (KHE) complicated by Kasabach–Merritt phenomenon (KMP) treated at our institution.

## Case Presentation

**Fig. 1:**
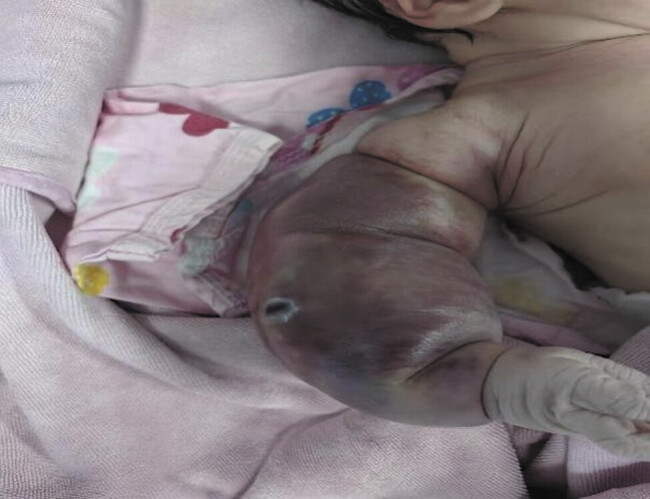
Postnatal clinical presentation of the right upper limb.

**Fig. 2:**
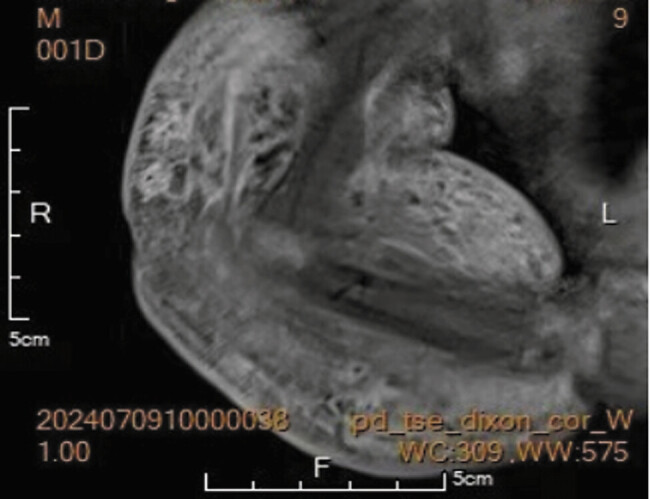
Magnetic resonance imaging findings of vascular tumor infiltration with hemorrhage.

**Fig. 3:**
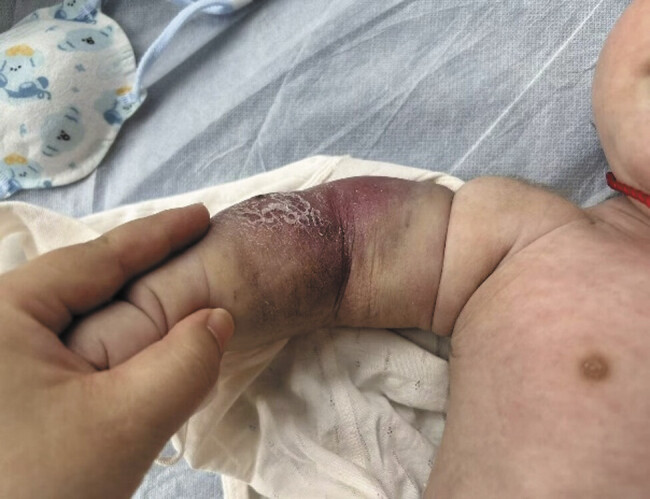
Clinical assessment at 2-month follow-up demonstrating significant tumor regression.

**Fig. 4:**
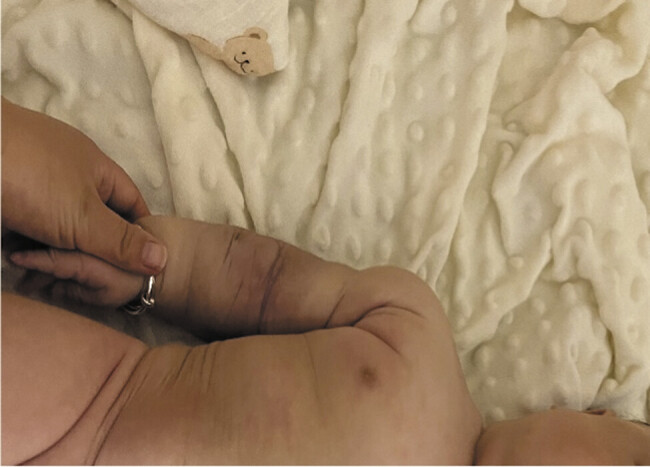
Radiological and clinical evaluation at 4-month follow-up confirming complete tumor resolution.

A male neonate, first-born of a singleton pregnancy delivered vaginally at 40 weeks' gestation with a birth weight of 3,150 g, had Apgar scores of 9 (deducted 1 point for cyanosis) at 1 minute and 10 at 5 minutes. Prenatal ultrasound performed 5 days prior to delivery revealed right upper limb thickening (subcutaneous tissue thickening with enhanced echogenicity). Postnatally, a dark red mass on the right upper limb was noted within 1 hour, prompting referral to pediatric surgery with a provisional diagnosis of KHE. Physical examination demonstrated a swollen, violaceous, firm mass with elevated skin tension, ill-defined borders, limited mobility, and blanchable discoloration upon pressure; distal finger mobility was preserved ( Fig. 1 ). Laboratory tests revealed hemoglobin 127 g/L, severe thrombocytopenia (platelets: 16 × 10 ^9^ /L), neutrophil ratio 60.3%, and hypofibrinogenemia (1.70 g/L). Magnetic resonance imaging (MRI) of the right upper limb confirmed vascular endothelial neoplasia with minimal hemorrhage ( Fig. 2 ), supporting a diagnosis of KHE complicated by KMP. Due to recent Bacillus Calmette–Guérin (BCG) vaccination, initial management excluded sirolimus and included topical tacrolimus, compression therapy, vitamin K, and corticosteroids. Platelet counts transiently improved to 23 × 10 ^9^ /L on day 2 but declined to 11 × 10 ^9^ /L on day 3, accompanied by worsening hypofibrinogenemia (0.72 g/L) and hemoglobin drop (111 g/L). After parental consent, oral sirolimus (0.25 mg/m ^2^ /day) was initiated, and the infant was transferred to the neonatal intensive care unit. Re-examination shows a drop in hemoglobin, suggesting bleeding. Two fibrinogen transfusions stabilized coagulation parameters. By day 13, platelets normalized (283 × 10 ^9^ /L), and sirolimus was continued alongside transitioning corticosteroids from intravenous to oral. On day 21, fever and diarrhea emerged but resolved with anti-inflammatory agents, rehydration, and symptomatic care. At discharge (day 27), platelets stabilized at 183 × 10 ^9^ /L, with marked tumor regression ( Figs. 3 and 4 ). Postdischarge, oral sirolimus and prednisone were maintained. Prophylactic trimethoprim-sulfamethoxazole (20 mg/kg twice daily, 3 days/week) was added at 2 months to prevent Pneumocystis infection. Three-month follow-up demonstrated sustained platelet stability (268–532 × 10 ^9^ /L), no infections, and significant clinical improvement. Ongoing monitoring confirms favorable outcomes.

## Discussion

KHE is a rare, locally aggressive borderline vascular tumor with a multifactorial pathogenesis. Current evidence suggests that KHE may be associated with mitogen-activated protein kinase pathway alterations caused by the GNA14 c.614A > T (p.Gln205Leu) mutation. Additionally, germline TP53 single-nucleotide variants in the context of coexisting oncogenic drivers may contribute to KHE development, while somatic second-hit mutations likely play a critical role in disease progression. 2 3

KHE has an estimated incidence of 0.7 per 10 million neonates, accounting for approximately 2% of all vascular tumors. Over 90% of cases manifest within the first year of life, with 60% presenting within the first month. Cutaneous or subcutaneous lesions typically appear as firm, violaceous nodules or plaques displaying heterogeneous pigmentation and ill-defined borders secondary to ecchymosis or telangiectasia. KHE can trigger KMP, a life-threatening coagulopathy initiated by abnormal vascular endothelial cell proliferation within KHE lesions. These cells sequester platelets, promoting platelet adhesion, aggregation, and activation. This process locally activates the coagulation cascade, leading to excessive fibrin deposition and microthrombus formation. Subsequent consumption of platelets and clotting factors exacerbates coagulopathy, culminating in disseminated intravascular coagulation and potential mortality. 2

The diagnosis of KHE requires a multidisciplinary approach integrating clinical evaluation, imaging studies, and histopathological examination. Prenatal ultrasound serves as a diagnostic modality, with some cases detectable during the third trimester of pregnancy. Postnatal magnetic resonance imaging is pivotal for distinguishing KHE from other vascular malformations, defining lesion extent, and assessing therapeutic response during follow-up. 3 The diagnosis of KHE involving the right upper limb, complicated by KMP, was confirmed postnatally through comprehensive evaluation, including physical examination, MRI, hematologic profiling (e.g., blood cell analysis), and coagulation function studies. Although biopsy remains the diagnostic gold standard, it was not performed in this case due to the potential to exacerbate the infant's consumptive coagulopathy.

A study involving six pediatric patients with superficial KHE treated with topical tacrolimus demonstrated an overall response rate of 100%, with no rebound growth of KHE observed at the final follow-up after treatment cessation. The therapeutic mechanism may be attributed to tacrolimus-mediated calcineurin inhibition, which reduces the production of T cell-derived cytokines and suppresses angiogenic factor synthesis, thereby impeding pathological vascular proliferation. 4

Glucocorticoids are employed in combination therapy for KMP to mitigate thrombocytopenia and suppress fibrinolysis. However, prolonged glucocorticoid use may result in transient growth impairment, heightened infection susceptibility, and behavioral changes. Gradual tapering and discontinuation are advised once clinical stabilization is achieved. 2

Sirolimus, an inhibitor of the mammalian target of rapamycin, exerts therapeutic effects by suppressing the mTOR/PI3K/AKT signaling pathway and downregulating vascular endothelial growth factor production. This dual mechanism effectively inhibits vascular endothelial cell proliferation and angiogenesis, establishing sirolimus as the first-line systemic therapy for KHE. 5 However, a case report documented disease recurrence in a pediatric patient following sirolimus treatment. 6 Due to the immunosuppressive effects of sirolimus, patients are at increased risk of upper respiratory tract infections, pneumonia, and cutaneous infections. Additionally, the potential for Pneumocystis pneumonia necessitates prophylactic administration of trimethoprim-sulfamethoxazole to mitigate infection risks. 7

A neonate presented with right upper limb KHE complicated by KMP. Given the patient's recent BCG vaccination, immediate sirolimus administration posed significant risks of immunosuppression-related complications, including disseminated tuberculosis (e.g., tuberculous meningitis). Therefore, topical tacrolimus was initiated as primary therapy at doses maintained within established safety thresholds. KHE lesions exhibited a significant clinical response to twice-daily topical application. 2 First-line therapies, including topical medications, compression therapy, and corticosteroids, failed to improve symptoms, prompting eventual sirolimus administration (0.25 mg/m ^2^ /day) to control KMP. TMP-SMX prophylaxis was deferred until 2 months of age due to the infant's immature acetyltransferase system, which elevates free sulfonamide levels and increases kernicterus risk. During hospitalization, transient fever and diarrhea resolved with anti-inflammatory and antidiarrheal therapies. At 2 months of age, TMP-SMX was initiated for infection prevention. Platelet transfusion was deliberately withheld in this patient. Platelets contain pro-angiogenic growth factors that may potentiate vascular lesion progression. Furthermore, transfused platelets can become sequestered within the lesion, precipitating acute expansion with concomitant activation and consumption of coagulation factors. 8 Thyroid function tests were not performed in this case. Routine thyroid screening will be systematically incorporated into the management protocol for subsequent patients. Follow-up confirmed sustained clinical stability, with no recurrence and age-appropriate developmental progress. Thyroid function tests were not performed in this case. Routine thyroid screening will be systematically incorporated into the management protocol for subsequent patients.

## Conclusion

KHE is a rare and complex vascular disorder often complicated by KMP. Prenatal ultrasound can detect KHE prenatally, while postnatal diagnosis is confirmed through clinical manifestations, MRI, hematologic and coagulation profiles, and histopathological examination. Timely treatment is critical, with current therapeutic options including local compression therapy, glucocorticoids, and oral medications such as sirolimus. Clinicians must tailor treatment strategies based on individual contraindications and disease severity. This case aims to provide clinical insights to guide evidence-based management of KHE and KMP.

## Notes

**Erratum:** This article was published with incorrect page numbers. The page numbers have been corrected subsequently with the publication of an erratum (DOI: 10.1055/a-2706-9632).

## References

[1] Croteau S E Liang M G Kozakewich H P Kaposiform hemangioendothelioma: atypical features and risks of Kasabach-Merritt phenomenon in 107 referrals J Pediatr 2013 162 01 142 147 22871490 10.1016/j.jpeds.2012.06.044 PMC3494787

[2] Ji Y Chen S Yang K Xia C Li L Kaposiform hemangioendothelioma: current knowledge and future perspectives Orphanet J Rare Dis 2020 15 01 39 32014025 10.1186/s13023-020-1320-1 PMC6998257

[3] Drolet B A Trenor C C III Brandão L R Consensus-derived practice standards plan for complicated Kaposiform hemangioendothelioma J Pediatr 2013 163 01 285 291 23796341 10.1016/j.jpeds.2013.03.080

[4] Zhang X Yang K Chen S Ji Y Tacrolimus ointment for the treatment of superficial kaposiform hemangioendothelioma and tufted angioma J Dermatol 2019 46 10 898 901 31373046 10.1111/1346-8138.15031

[5] Dang N Ren Y A case of superficial kaposiform hemangioendothelioma treated with oral propranolol combined with topical sirolimus Vasc Health Risk Manag 2024 20 251 254 38883398 10.2147/VHRM.S461505 PMC11180431

[6] Wang L Li J Wu C Case report: experience of a rare case of rebound of the Kasabach-Merritt phenomenon during sirolimus treatment in kaposiform hemangioendothelioma Front Pediatr 2022 10 949950 35990005 10.3389/fped.2022.949950 PMC9391052

[7] Kalbfell R Cohen-Cutler S Grisham E Infectious complications of vascular anomalies treated with sirolimus: a systematic review Pediatr Blood Cancer 2024 71 01 e30758 37933207 10.1002/pbc.30758

[8] Crary S E Hemostasis and thrombosis risks and management in vascular anomalies Hematology (Am Soc Hematol Educ Program) 2024 2024 01 718 723 39644036 10.1182/hematology.2024000597 PMC11665655

